# Meta-Analysis of Death and Myocardial Infarction in the DEFINE-FLAIR and iFR-SWEDEHEART Trials

**DOI:** 10.1161/CIRCULATIONAHA.117.030430

**Published:** 2017-12-11

**Authors:** Colin Berry, John D. McClure, Keith G. Oldroyd

**Affiliations:** 1British Heart Foundation Glasgow Cardiovascular Research Centre, Institute of Cardiovascular and Medical Sciences, University of Glasgow, Scotland (C.B., J.B.M.); 2West of Scotland Heart and Lung Centre, Golden Jubilee National Hospital, Clydebank, United Kingdom (C.B., K.G.O.).

**Keywords:** death, fractional flow reserve, instantaneous wave-free ratio, meta-analysis, myocardial infarction

In patients with coronary heart disease, revascularization can improve symptoms and, in certain high-risk subgroups, may improve prognosis. Coronary angiography provides anatomic information, and the physiological significance of a stenosis can be determined using fractional flow reserve (FFR). Decisions on the need for and mode of revascularization can be optimized using FFR. However, this process involves administering adenosine to induce hyperemia. Generally, this is well tolerated, but in some healthcare systems, adenosine is either not licensed, unavailable, or expensive, limiting the use of FFR-guided management.

Recently, alternative approaches to FFR have emerged, including resting indices such as Pd/Pa and instantaneous wave free ratio (iFR).^1,2^ Hybrid algorithms incorporating a resting index reduce the need for adenosine by ≈50% or a hybrid algorithm utilizing contrast FFR reduces adenosine use even further (~65%).^3^ These diagnostic approaches represent clinically useful advances provided health outcomes are not compromised.

The DEFINE-FLAIR trial (Functional Lesion Assessment of Intermediate Stenosis to Guide Revascularisation)^1^ and the iFR-SWEDE-HEART trial (Instantaneous Wave-free Ratio versus Fractional Flow Reserve in Patients with Stable Angina Pectoris or Acute Coronary Syndrome)^2^ compared iFR- versus FFR-guided management using binary cutoff values in both groups. The primary composite outcome of death, myocardial infarction (MI), and urgent revascularization at 12 months and the noninferiority designs were consistent across both trials. Overall, an iFR-guided strategy was associated with a lower use of revascularization, and the primary end point results of both trials met the prespecified noninferiority criteria. The numerically dominant component of the primary outcome was unplanned revascularization. The rationale of our study was to assess the risk of death and MI between the iFR- and FFR-guided groups in a pooled analysis of these trials.

Our objective was to undertake a meta-analysis of the pooled events for death and MI in the DEFINE-FLAIR and iFR-SWEDE-HEART trials. The principal summary measure was the risk ratio (95% confidence interval [CI] and *P* value) calculated for each study. Meta-analysis estimates were calculated from a random effects model using the REML method. Fixed effects analyses using the Cochrane-Mantel-Haenzel method produced near identical results (not shown). I^2^ was used to measure the consistency of the meta-analysis. The analysis was conducted with R (version 3.10) using the metaphor (https://CRAN.R-project.org/package=metafor) and rmeta (https://CRAN.R-project.org/package=rmeta) packages.

The study characteristics and results are summarized in the Table. In total, 160 deaths or MI events occurred in 4345 participants during the 12 months after randomization. Of these events, 90 occurred in the iFR group (n=2159), and 70 events occurred in the FFR group (n=2186) (hazard ratio, 1.30; 95% CI, 0.96−1.77; *P*=0.09). Considering the hazard ratio for death or MI, the lower CI limit crosses unity. The upper CI limit indicates that the risk of this adverse outcome could be ≤77% greater for iFR guidance compared with FFR guidance. No evidence of heterogeneity was found between the 2 studies (I^2^ was 0% and χ^2^ *P*>0.5 for all analyses and unplanned revascularizations were I^2^=16% and χ^2^ *P*=0.28). We identified a risk of bias in these trials because any coronary revascularization after 60 days was defined as unplanned, but this procedure (a primary outcome event) was ordered by a physician who may have had knowledge of the treatment group assignment because of the open-label trial design (DEFINE-FLAIR attempted to blind the treating clinician to whether iFR or FFR was performed, but this was not done in iFR-SWEDE-HEART).

In the DEFINE-FLAIR and iFR-SWEDE-HEART trials, we observed a numeric excess of death or MI events in the iFR compared with the FFR groups. Directional consistency exists for this outcome in both trials and also when considering death and MI as separate outcomes. Both trials have relevant design limitations. First, because of the concordance between iFR and FFR in 80% of patients, the randomized strategy could only influence outcome in 20% of trial participants, diluting the power of both studies to detect a clinically meaningful difference in outcomes. Second, in the context of other evidence, the discordance between iFR and FFR is greatest in stenoses of the left main and proximal coronary arteries,^4,5^ which is where revascularization may confer a survival advantage. The distribution of coronary disease in the trial participants has not been reported. Finally, the populations studied in both trials were at relatively low cardiovascular risk, with incidence of death, MI, and repeat revascularization at 1 year ≈50% of what was observed in the FAME trial (Fractional Flow Reserve versus Angiography for Guiding Percutaneous Coronary Intervention), highlighting the limited power for detecting any difference in clinically important health outcomes between the 2 strategies in the current trials.

In conclusion, in a pooled meta-analysis of the DEFINE-FLAIR and iFR-SWEDE-HEART trials, a numeric excess of death and MI events occurred in the iFR group that is not statistically significant and, therefore, hypothesis generating. Considering death and MI, iFR-guided management may not be noninferior to FFR-guided management. Further research seems warranted.

**Table.**
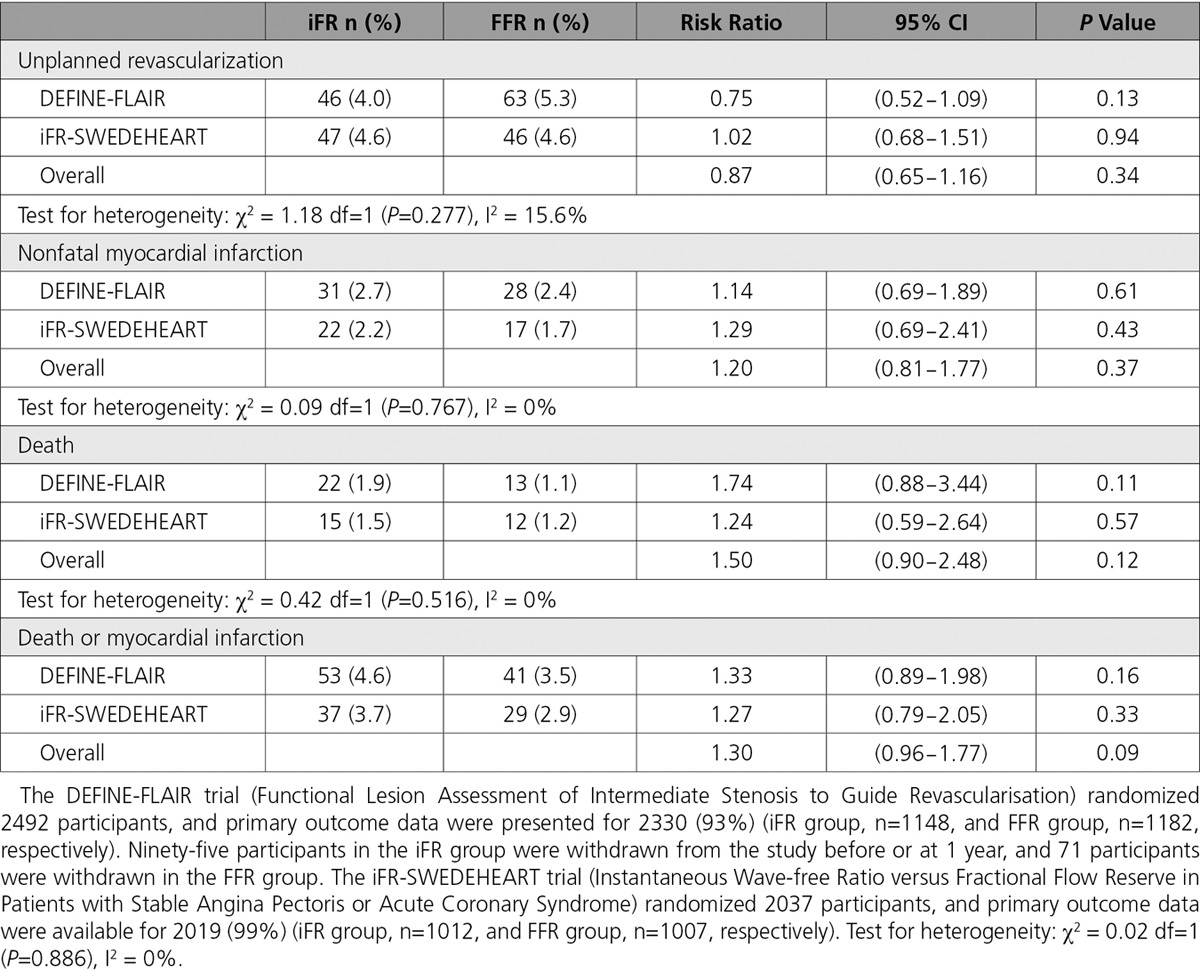
Unplanned Revascularization and Spontaneous Adverse Outcomes at 12 Months in DEFINE-FLAIR (Functional Lesion Assessment of Intermediate Stenosis to Guide Revascularisation) and iFR-SWEDEHEART (Instantaneous Wave-free Ratio versus Fractional Flow Reserve in Patients with Stable Angina Pectoris or Acute Coronary Syndrome) Trials

## Sources of Funding

This work was supported by the University of Glasgow and the British Heart Foundation (RE/13/5/30177, PG/14/97/31263). The funders had no involvement in the analysis.

## Disclosures

Dr Berry received a significant research grant and modest honoraria; and, based on an institutional agreement with the University of Glasgow, acted as a consultant to Abbott Vascular. The company had no involvement in any aspect of the manuscript. Dr Oldroyd received modest honoraria and has acted as a consultant to Abbott Vascular. Dr McClure reports no conflicts of interest.
